# Six‐Month Emergent Readmissions Following Hospitalization for Atrial Fibrillation Amid the Syrian Conflict: A Real‐World Observational Cohort Study

**DOI:** 10.1111/jce.16579

**Published:** 2025-01-13

**Authors:** Ibrahim Antoun, Alkassem Alkhayer, Alamer Alkhayer, Khaled Yazji, Riyaz Somani, G. André Ng, Mustafa Zakkar

**Affiliations:** ^1^ Department of Cardiovascular Sciences University of Leicester Leicester UK; ^2^ Faculty of Medicine University of Aleppo Aleppo Syria; ^3^ Faculty of Medicine University of Tishreen's Hospital Latakia Syria; ^4^ Department of Cardiology The View Hospital Qatar Qatar; ^5^ Department of Cardiology University Hospitals of Leicester NHS Trust, Glenfield Hospital Leicester UK; ^6^ Department of Research NIHR Leicester Biomedical Research Centre Leicester UK; ^7^ Department of Cardiac Surgery University Hospitals of Leicester NHS Trust, Glenfield Hospital Leicester UK; ^8^ Faculty of Medicine University of Damascus Damascus Syria

**Keywords:** atrial fibrillation, conflict, mortality, readmission, Syria

## Abstract

**Background:**

Atrial fibrillation (AF) is the most common arrhythmia worldwide. However, data regarding readmissions following index admission for AF in the developing world are not well described. This study assessed the rate, predictors, and trends of 6‐month readmission after index admission for AF in Syria.

**Methods:**

We included adult patients who had an index admission with AF to Latakia's tertiary center between July 2021 and November 2023. Patients were monitored for readmission for 6 months after index discharge. Data were taken from the patient's medical notes.

**Results:**

A total of 649 patients were included in the final analysis, of which 320 (49%) were readmitted to the hospital within 6 months following index admission. Cardiac causes were the most common cause of readmission in 76% of patients, of which 70% were AF. Readmitted patients had a higher median age (64 vs. 58; *p* = 0.001) and fewer males (49% vs. 36%; *p* = 0.001). In multivariate analysis, factors that independently increased 6‐month readmission risk were age ≥ 60 years (hazard ratio [HR]: 1.7, 95% CI: 1.4−2.2), females (HR: 2.2, 95% CI: 1.6−2.7), and congestive heart failure (CCF) (HR: 2.1, 95% CI: 1.4−2.6). Most cardiac readmissions (76%) happened during the first 60 days following index discharge.

**Conclusion:**

Almost half the patients were readmitted within 6 months after an index admission for AF. Females, CCF, and advancing age were independently associated with an increased risk of 6‐month readmission.

## 1 Introduction

Atrial fibrillation (AF) is the most common type of arrhythmia worldwide, and its prevalence in middle‐ to low‐income countries is underestimated [1]. AF in the developed world is well studied. Still, there is little data on AF management and demographics in the Middle East, with only four data registries [2]. AF‐related research in Arab countries contributed only 0.7% of AF research worldwide [3].

Syria has been suffering from a conflict since 2011. It has been deprived of healthcare funding and resources, particularly exacerbated during the cholera and COVID‐19 outbreaks [4, 5]. Therefore, less than 50% of its hospitals operate at usual performance, with more than half of its healthcare workforce forced to leave the country due to conflict [6]. AF management in the hospitals during the current economic and political turmoil is unclear, with a paucity of published inpatient figures and outcomes originating from Syrian healthcare. However, there has been a recent effort to validate the quality of life questionnaires in these Syrian AF patients [7]. In the context of the resource limitations, a real‐world depiction of the current AF care can help manage and allocate resources by recognizing remediable deficiencies and, more importantly, practical and reasonable solutions that could be enforced [8]. Although late advances in AF management have enhanced the AF burden and symptom control, readmission rates continue to increase and have been one of the primary sources of AF‐related financial constraints on healthcare economies around the world. Particularly for Syria, following up on patients after initial admissions related to AF is highly challenging due to limited resources and damaged infrastructure [6]. Readmission rates and predictors of readmission have been studied in the United States [9, 10, 11, 12], New Zealand, and Australia [13] but not in developing countries under conflict settings.

This study aimed to describe the characteristics of Syrian patients readmitted to Latakia's tertiary center within 6 months of an index admission for primary AF.

## 2 Methods

### 2.1 Design and Data Collection

This is a retrospective single‐center observational cohort study conducted at Tishreen's University Hospital in Latakia, Syria, from July 2021 to November 2023. The hospital is a large government‐operated public institution associated with Tishreen University, and it serves as the primary healthcare center for the city and the surrounding areas. The hospital has around 860 beds and provides free healthcare. On average, the hospital sees approximately 50 000−60 000 inpatients yearly, with an even more significant number of outpatients seeking care in various medical departments. The Cardiology Department at Tishreen University Hospital comprises around 10 general cardiology consultants. The department is well‐equipped to handle both emergency and elective cases of cardiovascular diseases, and it has access to state‐of‐the‐art cardiac diagnostic tools, including echocardiography and cardiac catheterization facilities. The study included patients > 18 years old who were treated for AF as the primary diagnosis during admission. Patients < 18 and those with missing data for sex and age were excluded. Patients were followed for 6 months after discharge from their initial admission to track readmissions. Data sources for the study included hospital electronic and paper records. The medical or cardiology consultant determined the causes of the index admission and readmission. The research reported in this article adhered to the Declaration of Helsinki. The study was conducted as part of an audit approved by the hospital board and involved prospective analysis of retrospectively collected anonymized data. Therefore, the ethical committee of Tishreen's University Hospital waived the need for consent (reference 325/A).

### 2.2 Outcomes

The primary outcome of our study was 6‐month readmissions and their etiology. A secondary analysis was performed to explore predictors of 6‐month readmissions.

### 2.3 Statistical Analysis

Continuous variables are expressed as median and interquartile ranges (IQR). Categorical variables are expressed as counts and percentages (%). Pearson's *χ* ^2^ or Fisher's exact test was used for categorical variables between groups. Student's *t*‐test was used to compare continuous variables.

Kaplan−Meier models and Cox regression were used to investigate the relationship between variables and the probability of readmission within 6 months. We hypothesized that specific demographics and comorbidities would affect the 6‐month readmission probability. Therefore, a base model was constructed to assess the incremental value of comorbidities significantly associated with 6‐month readmission. Statistically significant comorbidities in the univariate analysis were added to the multivariable analysis. A two‐sided *p* < 0.05 was considered statistically significant. Statistical analysis was performed using GraphPad Prism V10.3 for Mac (San Diego, CA, USA).

## 3 Results

### 3.1 Baseline Characteristics

Our study included 649 consecutive patients with an index admission with primary AF as between July 2021 and November 2023. Among these, 320 (49%) had an unplanned readmission within 6 months after discharge (Table 1). Compared to patients who were not readmitted within 6 months of discharge, the readmitted group had more females (74% vs. 45%; *p* < 0.001) group had older patients (median age 64 vs. 58 years; *p* < 0.001), more ischemic heart disease (IHD) (29% vs. 18%; *p* < 0.001), and more congestive heart failure (CCF) patients (26% vs. 13%; *p* < 0.001).

**Table 1:** Baseline characteristics of patients with 6‐months readmission versus no readmission after index admission with acute atrial fibrillation.

	Overall (649)	Readmission (*n* = 320)	No readmission (329)	*p* value
Demographics (median IQR or %)				
Age (years)	60 (54−68)	64 (54−71)	58 (53−64)	**< 0.001**
Male	387 (60%)	145 (45%)	242 (74%)	**< 0.001**
Cardiovascular comorbidities (%)				
Hypertension	216 (33%)	93 (33%)	128 (34%)	0.56
Ischemic heart disease	154 (24%)	83 (29%)	69 (18%)	**0.001**
Diabetes mellitus	152 (23%)	102 (36%)	59 (16%)	0.27
Cerebrovascular disease	122 (19%)	47 (17%)	75 (20%)	0.32
Congestive heart failure	116 (18%)	72 (26%)	48 (13%)	**0.001**
PCI within the last year	37 (6%)	23 (7%)	14 (4%)	0.13
Thyroid disease	23 (4%)	8 (3%)	16 (4%)	0.2
Other comorbidities (%)				
Anemia	106 (16%)	49 (15%)	57 (17%)	0.52
Dementia	50 (8%)	29 (9%)	21 (6%)	0.09
Active malignancy	44 (7%)	21 (7%)	23 (7%)	0.88
Chronic liver failure	50 (8%)	22 (7%)	28 (9%)	0.46
Chronic lung disease	66 (10%)	35 (11%)	31 (9%)	0.61
Chronic kidney failure	53 (8%)	23 (7%)	30 (9%)	0.14

*Note:* Bold values represents statistically significant results.

Abbreviations: IQR, interquartile ranges; PCI, primary coronary intervention.

### 3.2 Etiologies of 6‐Month Readmissions

**Figure 1:**
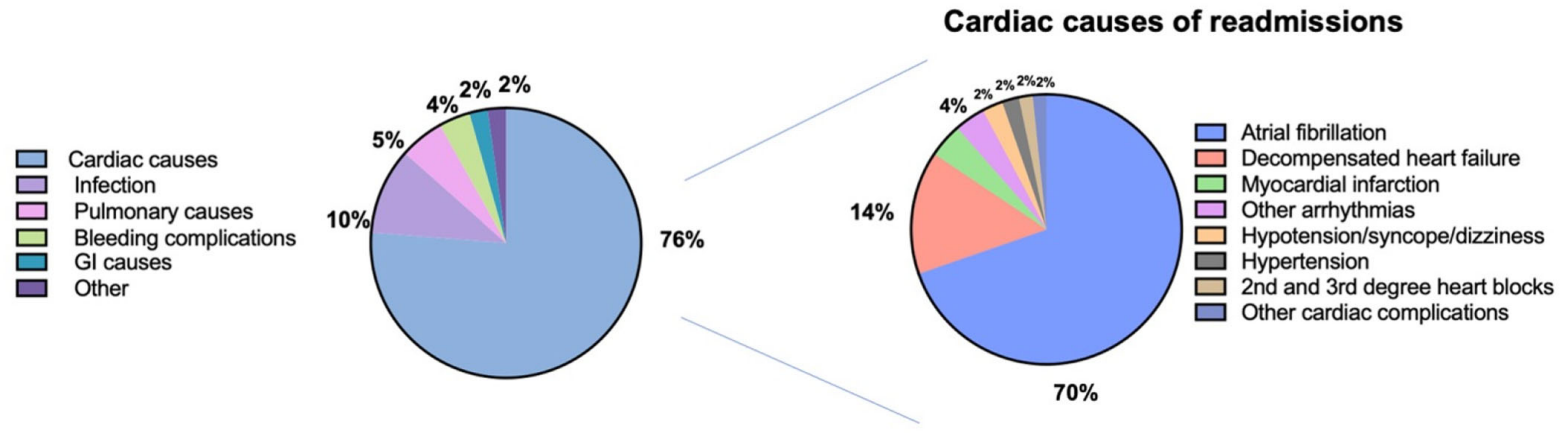
Etiologies of 6‐month readmissions after atrial fibrillation. GI, gastrointestinal.

Cardiac conditions were the most common cause of 6‐month readmission (76%). Of cardiac conditions, the most common condition was AF (70%), followed by CCF (15%) and myocardial infarction, along with arrhythmias other than AF (each at 4%). The most common noncardiac causes of readmission were infection (10%), pulmonary causes (5%), and bleeding events (4%) (Figure 1).

### 3.3 Predictors of 6‐Month Readmission

**Figure 2:**
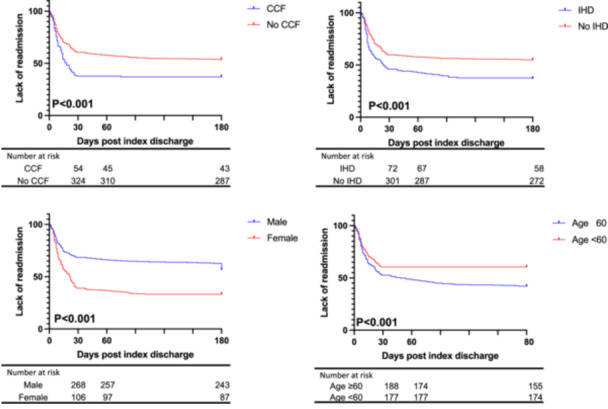
Kaplan−Meier analysis regarding predictors of 6‐month readmission following index admission for atrial fibrillation. CCF, congestive cardiac failure; IHD, ischemic heart disease.

We identified that the females were associated with an increased risk for readmission compared to the males at 6 months (67% vs. 43%, *p* < 0.001). Similarly, the presence of IHD was associated with an increased risk of admission at 6 months (42% vs. 56%, *p* = 0.001), patients ≥ 60 years at 6 months (58% vs. 40%, *p* < 0.001), DM at 6 months (42% vs. 57%, *p *= 0.005), and CCF at 6 months (40% vs. 61%, *p* < 0.001) as demonstrated by the Kaplan−Meier figures (Figure 2). Univariable Cox regression showed patients ≥ 60 years old (HR: 1.6, 95% CI: 1.3−2, *p* < 0.001), females (HR: 2, 95% CI: 1.6−2.4, *p *< 0.001), CCF (HR: 1.8, 95% CI: 1.3−2.5, *p* < 0.001), and IHD (HR: 1.8, 95% CI: 1.3−2.7, *p* = 0.001) were associated with increased probability of 6‐month readmission, while multivariate analysis showed that patients ≥ 60 years (HR: 1.7, 95% CI: 1.4−2.2, *p* < 0.001), females (HR: 2.2, 95% CI: 1.7−2.8, *p* < 0.001), and CCF (HR: 2.1, 95% CI: 1.6−2.7, *p* < 0.001) were independently associated with increased risk of 6‐month readmission (Table 2).

**Table 2:** Cox regression showing univariate and multivariable‐adjusted predictors of 6‐month readmission in patients who survived 6 months after index atrial fibrillation hospitalization.

	Univariable analysis		Multivariable analysis	
Presenting characteristic	HR (95% CI)	*p* value	HR (95% CI)	*p* value
Age ≥ 60 years (vs. < 60 years)	1.4 (1.3−1.8)	0.005	1.7 (1.4−2.2)	< 0.001
Females (yes vs. no)	1.4 (1.1−1.8)	0.01	2.2 (1.6−2.7)	< 0.001
Congestive cardiac failure (yes vs. no)	2 (1.5−2.8)	0.001	2.1 (1.4−2.6)	< 0.001
Ischemic heart disease (yes vs. no)	1.8 (1.3−2.7)	0.001	1.2 (0.9−1.8)	0.11
Diabetes mellitus (yes vs. no)	1.5 (1.1−2)	0.03		
Cerebrovascular disease (yes vs. no)	0.9 (0.6−1.6)	0.8		
Hypertension (yes vs. no)	1 (0.7−1.4)	0.83		
Index admission LOS (yes vs. no)	1.1 (0.9−1.2)	0.36		
Active thyroid disease (yes vs. no)	0.7 (0.3−1.5)	0.34		
PCI within the past year (yes vs. no)	1.3 (0.7−2.3)	0.30		
Chronic kidney disease (yes vs. no)	0.8 (0.5−1.2)	0.30		
Chronic liver disease (yes vs. no)	1.1 (0.7−1.7)	0.77		
Active malignancy (yes vs. no)	1.2 (0.7−2.2)	0.38		
Chronic lung disease (yes vs. no)	1.4 (0.9−2.1)	0.10		
Dementia (yes vs. no)	0.8 (0.5−1.6)	0.57		
Anemia (yes vs. no)	1.2 (0.8−1.6)	0.32		

Abbreviations: LOS, length of stay; PCI, primary coronary intervention.

### 3.4 Trends and Frequencies in 6‐Month Readmissions

**Figure 3:**
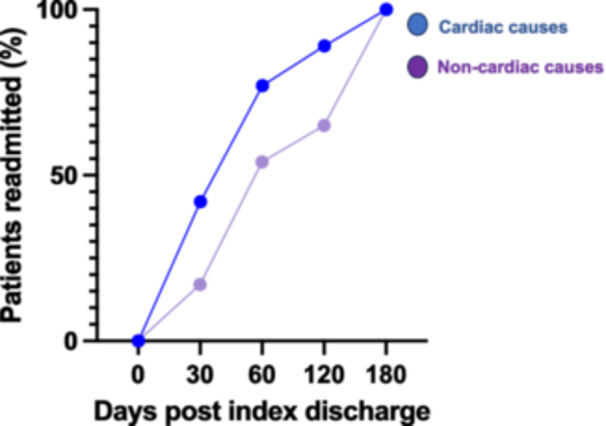
Comparison of readmission trends between cardiac and noncardiac causes. Fourty‐two percent of the readmissions due to cardiac causes happened within the first 30 days of index discharge, and 77% within the first 60 days following index discharge. Fifty‐two percent of the readmissions due to noncardiac causes happened within the first 60 days of index discharge.

Trends in readmissions within 6 months following index admission are shown in Figure 3. Most cardiac readmissions occurred during the first 60 days following index discharge (43% at 30 days and 76% at 60 days), while readmissions due to noncardiac causes peaked later, with 17% at 30 days and 53% at 60 days).

Of the patients readmitted, 233 (73%) were readmitted once, 48 (15%) were admitted twice, and 39 (12%) were readmitted thrice or more.

## 4 Discussion

This is the first study describing trends and predictors of 6‐month readmission following index admission with primary AF in the Middle East and Syria. It highlights several novel findings for our Syrian population. First, over half the patients were readmitted within 6 months of index admission, with AF being the most common cause. Second, CCF, females, and age ≥ 60 years were independently associated with an increased risk of 6‐month readmission. Third, most cardiac readmissions occurred within 60 days of index discharge.

As AF is known to have enormous implications on economies worldwide [14], recent studies have focused on many aspects of AF, including hospitalization, treatment patterns, and readmission rates [9]. Our 6‐month readmission rate of 49% exceeds rates reported from the US at 6 months of 38% [15]. Furthermore, our 6‐month readmission rate was higher than the reported rate of 2‐year readmission in Australia [16]. There was no developing world data to compare to. Our higher readmission rate can be explained by the conflict in Syria since 2011, which has primarily affected health infrastructure and caused an increased turnover of skilled staff and inadequate numbers of nurses and allied health professionals [17]. As only half the primary healthcare centers and hospitals are fully functional in Syria [17], managing risk factors and following patients presenting to hospitals with primary AF after discharge is challenging.

Also, access to medications during conflict has been challenging and should be addressed by international health organizations [18]. Patient engagement in high‐risk behaviors, mainly smoking, is correlated with unfavorable AF clinical outcomes [19]. For example, a recent Syrian study during the conflict demonstrated a smoking rate of 38% in 978 participants who had a mean age of 25 years, which was described as worrying [20]. Therefore, patient education and tackling these AF risk factors are essential to reducing the readmission burden and optimizing outcomes in resource‐depleted communities such as Syria.

Although there was no similar data before the conflict, supporting the Syrian healthcare system, including primary care, would help reduce the 6‐month readmission rate in Latakia and nationwide. Our cohort's most common readmission causes were cardiac, with AF being the most common. This was in keeping with previous literature [9, 11]. It is not uncommon for AF patients to be readmitted with the exact cause [21]. For example, in the Framingham study, only 10% of the AF patients were classified as having no recurrence in a community during the 2‐year follow‐up [22]. This can be because many AF patients have an underlying disease that is often unrecognized, together with the various coexisting comorbidities that could trigger AF. This will likely require subsequent early readmissions, such as in our cohort, with 15% and 12% having two and three readmissions or more, respectively, within the 6 months following index admission. The majority of cardiac readmissions occurred within the initial 60 days, possibly attributed to the relatively high prevalence of cardiovascular comorbidities in our patient cohort, leading to an increased risk of arrhythmias and CCF. Disparities in discharge planning, healthcare access, and care coordination can impact early readmission rates for these patients. Inadequate continuity of care and limited availability of specialized cardiac services may also have played a role in early readmissions in our study.

Our study demonstrated that a higher burden of cardiovascular disease (i.e., CCF and IHD) increased the risk of 6‐month readmission in keeping with the literature and came as no surprise [11, 14]. For example, older age was associated with an increased risk of 6‐month readmissions. This can be justified by the increased comorbidities burden and limited physiological reserve in these elderly populations, resulting in unfavorable outcomes. Females were at higher risk of 6 months readmission compared to males. The sex disparity in treatment utilization can explain this [23, 24] and provide an opportunity to pay attention to the underlying sociocultural mechanisms responsible for sex‐specific differences and identify barriers to effective AF treatment delivery in the developing world and Syria. If proven effective in further studies, our model can help identify patients at increased risk of readmission, which can guide patients' management during index admission. The early readmission rate with cardiac causes can be due to the challenges in post‐discharge follow‐up care, the lack of structured follow‐up programs, and limited access to outpatient services.

Additionally, socioeconomic disparities can impact patients' ability to follow through with treatment plans and attend follow‐up appointments. Limited financial resources may restrict access to necessary medications and interventions. It's essential to note that our study focused on participants from a developing country. Therefore, gathering more data from other developing countries is necessary to improve outcomes in communities with financial constraints.

## 5 Conclusion

The 6‐month readmission rate after index admission with primary AF was 49% in this Syrian center, with AF being the most common cause. Advancing age, females and CCF were independently associated with an increased probability of 6‐month readmission. Most cardiac readmissions with cardiac causes occurred within the first 60 days of index discharge. Our study advocates the importance of addressing risk factors in preventing AF‐related readmissions.

## 6 Limitations

Data collection was limited to a single tertiary care center in Latakia. This city was relatively less affected by the Syrian conflict than the other eastern and northern regions of Syria. Therefore, considering the significant variation in the quality and availability of hospital resources and staff, our findings may not apply to other areas or centers. Additionally, our analysis only included data routinely recorded in medical records and the number of patients who visited the hospital. Consequently, there may be other factors affecting mortality that have not been identified. This study did not investigate treatments during the initial admission or discharge rhythm, which could have influenced the results. Readmissions to centers other than those studied were not accounted for, potentially leading to an underestimation of the readmission rate.

## Author Contributions

I.A. designed the study, analyzed the data, and wrote the first draft of the manuscript. A.A. and A.A. managed data collection. K.Y., M.Z., G.A.N., and R.S. reviewed and edited the manuscript.

## Ethics Statement

The study was conducted as part of an audit approved by the hospital board and involved prospective analysis of retrospectively collected anonymized data. Therefore, the ethical committee of Tishreen's University Hospital waived the need for consent (reference 325/A).

## Consent

The authors have nothing to report.

## Conflicts of Interest

The authors declare no conflicts of interest.

## Data Availability

Data relating to this study are available upon reasonable request from the corresponding author.
